# CD2–CD58 axis orchestrates cytotoxic T lymphocyte function and metabolic crosstalk in breast cancer brain metastasis

**DOI:** 10.1002/ccs3.70040

**Published:** 2025-08-24

**Authors:** Guanyou Huang, Yigong Wei, Xiaohong Hou, Xin Jia, Yong Yu, Xu Li, Shanshan Yu

**Affiliations:** ^1^ Department of Neurosurgery The Second People's Hospital of Guiyang (Jinyang Hospital) Guiyang China; ^2^ Department of Oncology The Second People's Hospital of Guiyang (Jinyang Hospital) Guiyang China; ^3^ Department of Neurocritical Care The Second People's Hospital of Guiyang (Jinyang Hospital) Guiyang China

**Keywords:** breast cancer brain metastasis, CD2‐CD58 axis, CD8^+^ Effector T cell, immunometabolic crosstalk, single‐cell transcriptomics, tumor metabolic reprogramming

## Abstract

This study investigates the impact of the CD2–CD58 signaling axis on effector T cell function and tumor metabolic crosstalk in breast cancer brain metastasis (BCBM) using single‐cell transcriptomic analysis. scRNA‐seq data analysis revealed the critical role of CD2–CD58 signaling between CD8^+^ T cells and tumor cells in BCBM. Functional assays demonstrated that CD2 knockdown inhibited cytotoxic T lymphocyte (CTL) proliferation, activation, and cytotoxicity, leading to impaired tumor cell recognition and enhanced proliferation, migration, and invasion. In vivo studies showed that CD2‐deficient CTLs promoted tumor growth and brain metastasis while affecting metabolic reprogramming by altering key enzyme expressions in pyrimidine biosynthesis and arginine metabolism pathways. The findings suggest that CD2 enhances CTL function against tumor cells and influences their metabolic states, highlighting the role of CD2 in remodeling the brain metastatic microenvironment in breast cancer.

## Graphical abstract

Mechanistic model of CD2–CD58 axis‐mediated immune‐metabolic crosstalk between effector T Cells and tumor cells in the BCBM microenvironment. Created in BioRender.

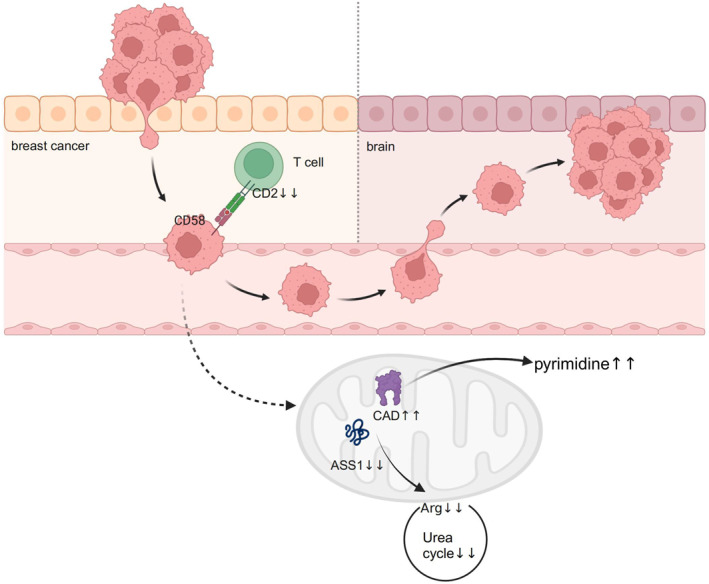

## 1 INTRODUCTION

Breast cancer ranks as the most frequently diagnosed malignancy among women globally and remains a leading cause of cancer‐related mortality. Brain metastasis represents one of the most devastating forms of distant spread, occurring in approximately 10%–30% of patients, with particularly high incidence in HER2‐positive and triple‐negative subtypes.^1^, ^2^, ^3^ Due to the restrictive nature of the blood–brain barrier (BBB) and the distinct immunometabolic environment of the brain, metastatic lesions exhibit marked resistance to conventional therapies, resulting in poor prognosis with median survival ranging from 6 to 12 months.^4^, ^5^ The tumor immune microenvironment (TIME) is critical in developing brain metastases. The degree of effector T cell infiltration, their activation state, and the extent of functional exhaustion directly influence immune control.^6^, ^7^, ^8^ With the advancement of immunotherapy, restoring T cell effector function and elucidating their interaction with the metastatic microenvironment have become key directions and potential breakthroughs in breast cancer brain metastasis (BCBM) research.

CD8^+^ effector T cells (cytotoxic T lymphocytes, CTLs) serve as key mediators of antitumor immunity by recognizing tumor cells through MHC class I molecules and inducing apoptosis through the release of perforin‐1 (PRF1), granzyme B (GZMB), and IFN‐γ. IL‐2 secretion further supports CTL proliferation and memory formation.^9^, ^10^, ^11^ However, in the immunosuppressive microenvironment of BCBM, CTLs frequently exhibit functional exhaustion, characterized by upregulation of immune checkpoints, such as PD‐1 and TIM‐3, reduced cytotoxic capacity, and suppression by inhibitory molecules (e.g., TGF‐β and IDO) and immunosuppressive cells (e.g., Tregs and MDSCs).^12^, ^13^, ^14^ In addition, tumor cells compete with CTLs for key metabolic substrates, restricting nutrient availability and further exacerbating T cell dysfunction.^15^, ^16^, ^17^ Recent studies suggest that specific signaling pathways and metabolic disruptions may drive CTL exhaustion, but the underlying mechanisms in BCBM remain poorly understood.^18^, ^19^ Unraveling the connections between CTL exhaustion and metabolic imbalance may uncover novel therapeutic targets for improving immunotherapy efficacy in BCBM.

CD2 is a critical adhesion and costimulatory molecule expressed on the surface of T cells. By binding to its ligand CD58, CD2 stabilizes the immunological synapse and enhances T cell receptor (TCR) signaling, thereby promoting effective T cell activation.^20^, ^21^ The CD2–CD58 signaling axis plays a central role in T cell activation, enhancement of cytotoxic function, and memory maintenance, while also modulating immune responses through pathways such as PI3K/AKT.^22^, ^23^, ^24^ Emerging evidence suggests that low CD2 expression may be associated with CTL exhaustion; however, whether CD2 regulates the metabolic crosstalk between CTLs and tumor cells remains unknown.^24^ Leveraging single‐cell transcriptomic technologies combined with functional assays to elucidate the role of the CD2–CD58 axis in tumor immunity and metabolic regulation may offer novel insights into the mechanisms driving BCBM.

Metabolic reprogramming is a key mechanism by which tumor cells adapt to their microenvironment to sustain proliferation and metastatic potential, particularly in the context of BCBM.^25^, ^26^, ^27^ Tumor cells gain a survival advantage in the brain by upregulating glycolysis, enhancing pyrimidine biosynthesis, and altering amino acid metabolism. Critical enzymes, such as carbamoyl‐phosphate synthetase 2/aspartate transcarbamylase/dihydroorotase (CAD) in pyrimidine synthesis and ASS1 in urea cycle regulation, have been implicated in this process.^28^, ^29^ Simultaneously, CD8^+^ T cell function relies heavily on adequate metabolic support, requiring substantial energy and nutrient availability during activation.^15^, ^30^ Within the tumor microenvironment (TME), nutrient competition imposed by tumor cells leads to metabolic restriction and functional impairment of CTLs, ultimately promoting their exhaustion.^31^, ^32^ Current research increasingly focuses on the metabolic interactions between tumor cells and CTLs, highlighting their role in immune regulation. In this context, we propose that CD2 regulates CTL activation and may influence their metabolic coupling efficiency, thereby participating in tumor metabolic reprogramming. This mechanism remains largely unexplored and may represent a novel avenue for advancing BCBM research.

Based on the above background, the present study aimed to systematically investigate the role of the CD2–CD58 signaling axis in regulating CTL function and its metabolic interaction with tumor cells during BCBM. Single‐cell transcriptomic data (GSE186344) from the gene expression omnibus (GEO) database were first analyzed to identify potential CD2–CD58 signaling activity between CTLs and tumor cells within brain metastatic lesions. Subsequently, a functional CTL model was established by lentiviral‐mediated CD2 knockdown. Through a combination of flow cytometry, ^3^H‐Thymidine (^3^H‐TdR) incorporation, cytotoxicity assays, and in vivo mouse experiments, we comprehensively evaluated the effects of CD2 on CTL activation, proliferation, and its capacity to recognize, induce apoptosis in, and suppress the metastatic potential of breast cancer cells. In parallel, LC–MS and western blot analyses were conducted to assess the expression of key enzymes involved in pyrimidine biosynthesis and the urea cycle in MDA‐MB‐231 cells and tumor tissues, thereby elucidating the role of CD2 in tumor metabolic reprogramming. By integrating immunological and metabolic perspectives, the study provides new insights into the regulatory mechanisms of CD2 and advances understanding of BCBM pathogenesis. Furthermore, the findings establish a theoretical foundation for considering CD2 as a potential biomarker and therapeutic target in breast cancer brain metastasis immunotherapy.

## 2 MATERIALS AND METHODS

### 2.1 Bioinformatics analysis of public single‐cell datasets

Single‐cell RNA sequencing (scRNA‐seq) data were obtained from the GEO (https://www.ncbi.nlm.nih.gov/geo/) under accession number GSE186344, which includes two brain metastatic samples from breast cancer patients. Data preprocessing, quality control, and normalization were conducted utilizing the Seurat package in R. Cells were filtered based on the following thresholds: nFeature_RNA >200 and < 5000; nCount_RNA >1000 and < 20000; and mitochondrial gene content <15%. A total of 18,472 high‐quality cells were retained for downstream analysis. Dimensionality reduction was performed using principal component analysis (PCA) on the top 2000 highly variable genes (HVGs). The number of principal components (PCs) selected for clustering was determined using ElbowPlot and JackStrawPlot. Cell clustering was carried out using Seurat's FindClusters function (resolution = 1), followed by visualization with Uniform Manifold Approximation and Projection (UMAP). Cluster‐specific marker genes were identified with Seurat's built‐in tools, and cell types were annotated using the SingleR package in combination with the CellMarker database. Target cell subsets were reclustered for higher‐resolution analysis. To explore intercellular communication, we specifically focused on CD8^+^ T cells and their interaction networks with other cell types, particularly tumor cells. Special attention was given to the CD2–CD58 signaling axis to investigate its potential regulatory role within the TME.

### 2.2 Cell isolation, processing, and coculture

Human whole blood from healthy donors was purchased from IPHASE (Suzhou, China). CD8^+^ T cells were isolated using magnetic bead‐based flow sorting with anti‐CD8 microbeads (11333D, Thermo Fisher Scientific, USA). Cell purity was assessed using a BD FACSymphony™ A5 cell analyzer, and CD8^+^ T cell purity exceeded 95%. Isolated CD8^+^ T cells were cultured in RPMI 1640 medium (A4192301) supplemented with 10% fetal bovine serum (FBS; A5670801) and 1% penicillin–streptomycin (15140148), all purchased from Thermo Fisher Scientific (USA). MDA‐MB‐231 breast cancer cells were cultured in DMEM medium supplemented with 10% FBS and 1% penicillin–streptomycin, maintained at 37°C in a humidified atmosphere with 5% CO_2_. Cells were harvested during the logarithmic growth phase using 0.25% trypsin digestion, washed with phosphate‐buffered saline (PBS), and passaged accordingly.^33^

Lentiviral vectors encoding sh‐NC or sh‐CD2 were constructed using the pLenti6/BLOCK‐iT™‐DEST system (K494300, Thermo Fisher Scientific) and packaged in HEK‐293T cells (CRL‐11268, ATCC). Viral supernatants were harvested 48 h post‐transfection and concentrated by Genechem (Shanghai, China). CD8^+^ T cells at ∼50% confluency were infected with lentivirus at an MOI of 5. The target sequences were as follows: sh‐NC: 5′‐CCTAAGGTTAAGTCGCCCTCG‐3′, sh‐CD2‐1: 5′‐GCCAGATGTGTGAGCCAGGAA‐3', and sh‐CD2‐2: 5′‐CCACCCACTTACCTTATGTCA‐3'. After 48 h, RT‐qPCR and western blot were performed to evaluate transduction efficiency, with sh‐CD2‐2 achieving the most pronounced suppression and selected for subsequent experiments (Supporting Information S1: Figure S1 A,B).

CD8^+^ T cells were activated with anti‐CD3/CD28 beads (11132D, Thermo Fisher Scientific, USA) for 15 min at 48 h postinfection.^34^ The expression of CD2 was then measured using RT‐qPCR and western blot to verify CTL activation. Compared with unstimulated CD8^+^ T cells, the CD2 mRNA and protein levels were significantly upregulated in antibody‐activated CD8^+^ T cells (CTL group), indicating successful activation (Supporting Information S1: Figure S1C,D). Cells were divided into three groups: CTL (activated CD8^+^ T cells), sh‐NC CTL (sh‐NC‐infected and activated), and sh‐CD2 CTL (sh‐CD2‐infected and activated).

Following activation, CTLs from each group were cocultured with MDA‐MB‐231 breast cancer cells at a ratio of 1:5 (CTL: tumor cells) for 24 h to simulate the metabolic interactions between CTLs and tumor cells in the TME.^35^, ^36^

### 2.3 RT‐qPCR analysis

Total RNA was extracted using the Cell RNA Isolation Kit (12183020, Thermo Fisher Scientific). For reverse transcription, 1 μg of total RNA was converted into cDNA using the First Strand cDNA Synthesis Kit (K1622, Fermentas). Quantitative PCR was performed using the BeyoFast™ SYBR Green One‐Step qRT‐PCR Kit (D7268S, Beyotime) on the ABI PRISM 7500 RT‐PCR System (Applied Biosystems, Thermo Fisher Scientific). All reactions were conducted in triplicate. Relative mRNA expression levels were calculated using the 2^−ΔΔCt^ method. GAPDH served as the internal reference gene. Reactions were run on the StepOnePlus RT‐qPCR System (Applied Biosystems). Primer sequences are listed in Supporting Information S1: Table S1.

### 2.4 Western blot

Total cellular proteins were extracted using RIPA lysis buffer (P0013C, Beyotime), and protein concentrations were determined using the BCA Protein Assay Kit (P0012, Beyotime). Equal amounts of protein (50 μg per sample) were separated on 8%–14% SDS‐PAGE gels and transferred to PVDF membranes through wet transfer. Membranes were blocked and incubated overnight with primary antibodies diluted in TBST: CD2 (1:1000, ab131276), CD58 (1:1000, ab308459), PRF1 (1:500, ab97305), GZMB (1:1000, ab134933), ASS1 (1:20000, ab170952), and CAD (1:1000, ab40800), all from Abcam (USA). After washing, membranes were incubated for 1 h at room temperature with an HRP‐conjugated secondary antibody (1:2000, ab6721, Abcam). Signals were visualized using ECL substrate (P0018S, Beyotime) and imaged with the Odyssey system (LI‐COR Biosciences). Band intensities were quantified using the Image Studio Lite software v5.2.5. GAPDH was used as a loading control.

### 2.5 Proliferation assay of CD8^+^ T cells

^3^H‐TdR Incorporation Assay: Activated CD8^+^ T cells (1 × 10^5^ cells/well) were seeded into 96‐well plates, and 1 μCi of ^3^H‐TdR (TRK120, PerkinElmer) was added to each well. Cells were incubated at 37°C in a 5% CO_2_ incubator for 6 h. After incubation, cells were harvested using a cell harvester and radioactivity was measured using a liquid scintillation counter to evaluate DNA synthesis and proliferation capacity.

CFSE Labeling Assay: CD8^+^ T cells were labeled with CFSE dye (HY‐D0056, MedChemExpress). After preparing the working solution, cells were incubated with the dye, washed twice with PBS, and cultured under standard conditions. Cells were collected and analyzed at designated time points to assess proliferation based on CFSE dilution.

### 2.6 Flow cytometry analysis

Apoptosis was detected using the Annexin V‐FITC/propidium iodide (PI) dual staining method. Cells were collected in 15 mL centrifuge tubes and centrifuged at 800 × g to remove the supernatant. The cell pellets were washed twice with PBS, then resuspended in 500 μL of binding buffer according to the instructions of the Annexin V‐FITC Apoptosis Detection Kit (556547, BD Biosciences). Next, 5 μL of FITC‐labeled Annexin V and 5 μL of PI were added in the dark, mixed gently, and incubated for 15 min at room temperature. Apoptotic cells were then analyzed using a BD FACSCalibur flow cytometer. Cells positive for Annexin V‐FITC staining were considered apoptotic. Each experiment was performed in triplicate.

### 2.7 Coimmunoprecipitation (Co‐IP)

CTLs and MDA‐MB‐231 cells were collected from the coculture system and lysed in strong RIPA buffer (P0013B, Beyotime) containing protease inhibitors (04693116001, Roche). Lysates were centrifuged at high speed at 4°C for 10 min, and supernatants were collected. For mouse tumor tissues, samples were homogenized in prechilled PBS, followed by lysis in RIPA buffer and incubation on ice for 30 min with intermittent mixing. After centrifugation, the supernatant was collected. Cell or tissue lysates were incubated with anti‐CD2 antibody (1:100, ab131276, Abcam, USA) at 4°C with gentle rotation for 12 h. After incubation, Protein A/G magnetic beads (HY‐K0202, MedChemExpress) were added to capture the antibody–antigen complexes. Beads were washed thoroughly, and bound protein complexes were eluted and analyzed using western blot to detect the CD58 expression. IgG was used as the negative control.

### 2.8 Assessment of CTL adhesion to tumor cells

To assess adhesion efficiency, activated CD8^+^ T cells were cocultured with MDA‐MB‐231 cells at a 1:5 ratio in RPMI 1640 medium containing 10% FBS for 24 h. Anti‐CD3/CD28 activation beads were subsequently added to enhance CTL activity. Cell–cell interactions were visualized under a light microscope (Leica Microsystems), and the percentage of conjugated CTLs was calculated to quantify adhesion efficiency.

### 2.9 Cell proliferation assay by cell counting kit‐8 (CCK‐8)

Cell proliferation was assessed using the CCK‐8 assay kit (WH1199, Weiao Biotech). Log‐phase cells were seeded into 96‐well plates at 5 × 10^4^ cells/mL (100 μL/well) and incubated for 24, 48, 72, and 96 h in DMEM containing 10% FBS. At each time point, the medium was removed and 10 μL of CCK‐8 solution was added. Plates were incubated at 37°C for 2 h, and absorbance was measured at 450 nm using a Multiskan FC microplate reader (51119080, Thermo Fisher Scientific). Cell proliferation rate (%) was calculated as: Proliferation (%) = [(A_control_ − A_experiment_)/A_control_] × 100%. Each group was tested in triplicate, and all experiments were repeated independently three times.

### 2.10 Transwell assay

Transwell assays were performed to evaluate the migration and invasion abilities of MDA‐MB‐231 cells. For migration, 1 × 10^5^ cells suspended in serum‐free medium were seeded into the upper chamber of Transwell inserts (8 μm pore size; 3422, Corning). The lower chamber contained RPMI 1640 medium with 10% FBS as a chemoattractant. After 24 h of incubation, cells remaining on the upper surface were removed with a cotton swab. Migrated cells on the underside were fixed, stained with crystal violet, and counted under a light microscope. For invasion analysis, Matrigel (354234, Corning) was precoated on the upper surface of the insert membrane. Subsequent procedures were identical to those in the migration assay, and invading cell numbers were used to quantify invasive capacity.

### 2.11 Animal model construction

Female nonobese diabetic/severe combined immunodeficient (NOD‐SCID) mice (6–8 weeks old, 18–22 g; strain code 394, Charles River) were maintained under specific pathogen‐free conditions (24°C, 55% ± 5% humidity, and 12 h light/dark cycle) with free access to food and water. All animal experiments were conducted in accordance with protocols approved by the Institutional Animal Care and Use Committee (IACUC) (Approval No. 2403172). To establish a BCBM model, 2 × 10^5^ luciferase‐labeled MDA‐MB‐231 cells in 0.1 mL PBS were injected into the heart's left ventricle. A subcutaneous xenograft model was generated by injecting 5 × 10^6^ MDA‐MB‐231 cells into the mice's right flank (subcutaneously).^37^ One week after tumor cell injection, 5 × 10^7^ CTLs transduced with either sh‐NC or sh‐CD2 were administered through tail vein injection.^38^ Mice were randomized into three groups: model, sh‐NC, and sh‐CD2. When tumors reached ∼15 mm in diameter, mice were euthanized and tumor tissues were harvested and stored at −80°C for further analysis. All procedures were conducted strictly with ethical standards for animal research.^39^

### 2.12 Bioluminescence imaging (BLI)

To monitor tumor progression and metastasis, mice were intraperitoneally injected with D‐luciferin potassium salt (150 mg/kg; HY‐12591B, MedChemExpress) and imaged using the IVIS LUMINA XRMS system (PerkinElmer, USA). At the endpoint, mouse brains were harvested for metastasis evaluation.

### 2.13 Tumor volume and weight measurement

In conscious or lightly anesthetized mice, the longest diameter (length) and shortest perpendicular diameter (width) of tumors were measured every other day. Tumor volume was calculated using the formula: Tumor volume (mm^3^) = (Length × Width^2^)/2. At the endpoint, mice were euthanized and tumors were carefully excised and weighed immediately after dissection.

### 2.14 TUNEL staining

TUNEL staining was performed on tumor tissue sections using the DeadEnd Fluorometric TUNEL System (Promega). Briefly, tissue sections were fixed with 4% formaldehyde, permeabilized in PBS containing 20 μg/mL proteinase K (P2308, Sigma‐Aldrich) and 0.2% Triton X‐100 (T9284, Sigma‐Aldrich). Slides were then incubated with the TdT reaction mixture at 37°C for 60 min, followed by mounting with a DAPI‐containing mounting medium (F6057, Sigma‐Aldrich). Fluorescent signals were visualized using a confocal microscope (Axio Imager A1; Carl Zeiss Ltd.) equipped with a fluorescein‐DAPI filter set (excitation: 340–380 nm; emission: 435–485 nm). All images were acquired under identical exposure settings and processed using the AxioVision Rel. 4.8 software.

### 2.15 Immunohistochemical (IHC) staining

Tumor tissue sections were initially fixed in 10% neutral buffered formalin, followed by paraffin embedding and sectioning at 4 μm thickness. After deparaffinization and antigen retrieval, sections were incubated with primary antibodies, including anti‐Ki67 (0.1 μg/mL, ab15580, Abcam) and cleaved Caspase‐3 (Asp175, 1:400, #9661, Cell Signaling Technology). Subsequently, the sections were stained with HRP‐conjugated goat antirabbit IgG secondary antibody (1:1000, ab6721, Abcam). Color development was performed using a DAB detection system (BL734A, Biosharp), and staining results were examined under a microscope. Positive cells for Ki67 and Caspase‐3 were counted using the ImageJ software, and the proportion of positively stained cells per field was calculated to assess protein expression levels.

### 2.16 Hematoxylin and eosin (H&E) staining

To assess metastatic lesions in specific brain regions, mice were anesthetized and perfused with 50 mL PBS followed by 25 mL of 4% paraformaldehyde (BL‐G002, Sbjbio). Brains were postfixed overnight at 4°C in 4% paraformaldehyde, then stored in PBS containing 0.1% sodium azide. The cerebellum, hippocampus, and striatum (coronal sections at −6.12, −1.82, and 0.5 mm relative to bregma) were paraffin‐embedded and sectioned at 4 μm thickness, followed using H&E staining. Tissue sections were deparaffinized in xylene for 10 min, rehydrated in a series of ethanol solutions (100% ethanol for 3 min, 96% ethanol for 3 min, and 70% ethanol for 3 min), and then rehydrated in running tap water for 1 min. Sections were stained with hematoxylin solution (G1005‐100ML, Servicebio) for 10 min, differentiated in 1% hydrochloric acid in 70% ethanol for 20 s, and blued in 1% ammonia water for 10 s. Subsequently, sections were stained with eosin solution (G1005‐100ML, Servicebio) for 2 min. Slides were dehydrated through graded alcohols (70%, 96%, and 100% ethanol for 3 min each), cleared in dimethylbenzene for 4 min, and finally mounted with neutral resin. Images were captured by light microscopy, and metastatic areas were quantified using ImageJ (v1.29x, NIH) from 10 random visual fields per brain region. Tumor burden was expressed as the ratio of tumor area to total tissue area.

### 2.17 LC–MS analysis

Tumor cells were digested with trypsin and neutralized using a medium containing 10% FBS. After centrifugation at 500 × g for 5 min, the supernatant was discarded and cells were washed with 0.5 mL prechilled normal saline. Cells were counted, and 1.5 × 10^6^ cells were collected and lysed in 80% methanol (MFCD00004595, Merck). The lysates were centrifuged at 12,000 × g for 10 min at 4°C, and the supernatant was collected for further analysis. For tumor tissues, approximately 20 mg of sample was homogenized in 200 μL H_2_O using five ceramic beads. Metabolites were extracted by adding 800 μL of methanol/acetonitrile (1:1, v/v; methanol: 32213‐1L, Sigma; acetonitrile: 34851‐1L, Sigma). Samples were vortexed (30 s), sonicated (10 min), and centrifuged at 14,000 × g for 15 min at 4°C. The supernatant was collected for LC–MS analysis. LC–MS analysis was performed to detect pyrimidine metabolites (thymidine triphosphate (TTP) and cytidine triphosphate (CTP)) and arginine (Arg) levels. High‐purity standards were used for calibration, including Arg (MFCD00004595, Merck), TTP (T7004, Sigma‐Aldrich), and CTP (C1506, Sigma‐Aldrich). All experiments were repeated three times to ensure data accuracy and reliability.

### 2.18 Statistical analysis

Statistical analyses were performed using the R software (version 4.2.1, R Foundation for Statistical Computing, Vienna, Austria) and GraphPad Prism (version 10.1.2). Quantitative data were expressed as mean ± standard deviation (SD). Normality and homogeneity of variance were assessed prior to analysis. For datasets meeting parametric assumptions, one‐way analysis of variance (ANOVA) was performed, followed using Tukey's post hoc test for multiple comparisons. Differences were considered statistically significant at *p* < 0.05.

## 3 RESULTS

### 3.1 Single‐cell transcriptomic analysis reveals the key role of CD2/CD58‐mediated T cell‐tumor cell interactions in BCBM

**FIGURE 1:**
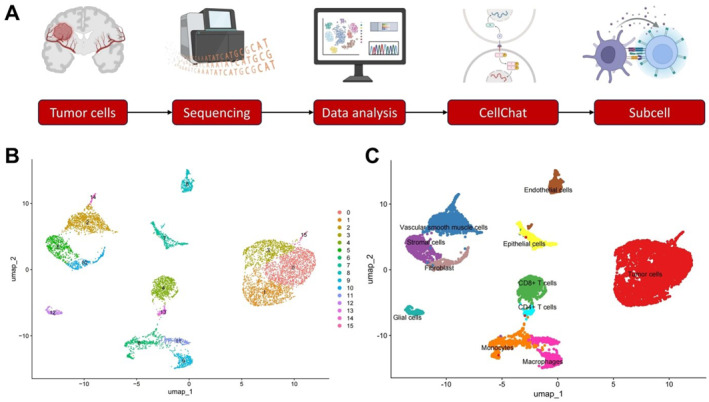
Single‐cell atlas of BCBM. (A) Bioinformatics workflow. Single‐cell RNA sequencing was performed on tumor tissues derived from BCBM. The data were subjected to sequential quality control, cell annotation, cell–cell communication analysis, and T cell subtype characterization. Created in BioRender; (B) uniform manifold approximation and projection analysis identified 16 distinct cell clusters; (C) the 16 clusters were annotated into 11 major cell types based on canonical marker gene expression. BCBM, breast cancer brain metastasis.

We first performed scRNA‐seq analysis of the TME in BCBM to identify key effector T cell subsets and their characteristic gene expression profiles. The overall workflow is illustrated in Figure 1A, including dataset selection, preprocessing, cell type annotation, and subsequent analyses. Using Seurat for data processing, a total of 18,472 high‐quality single cells were retained for downstream analysis (Supporting Information S1: Figure S2A), ensuring sufficient resolution for the identification of cellular heterogeneity. Correlation analysis revealed a positive correlation between nCount_RNA and nFeature_RNA (*r* = 0.93) and a negative correlation with percent.mt (*r* = −0.21), indicating robust data quality (Supporting Information S1: Figure S2B). HVGs were identified from the filtered cells, and the top 2000 HVGs were selected for downstream analyses (Supporting Information S1: Figure S2C). Using the JackStrawPlot function, we visualized the significance distribution of the top 20 PCs. PCs with smaller *p*‐values typically represent components that capture substantial biological variability, especially from the HVGs. The top 20 PCs had *p*‐values <0.05, indicating statistical significance (Supporting Information S1: Figure S2D). The ElbowPlot showed an inflection point at the 10th PC, suggesting it as the cutoff for downstream dimensionality reduction (Supporting Information S1: Figure S2E). Heatmaps for the top two PCs were generated using the DimHeatmap function (Supporting Information S1: Figure S3A), and the top genes contributing to PC1 and PC2 were also identified (Supporting Information S1: Figure S3B). Furthermore, expression characteristics of canonical marker genes across different cell clusters were visualized using feature plots, bubble plots, and violin plots (Supporting Information S1: Figure S3C–E).

UMAP‐based clustering analysis grouped the single cells into 16 distinct clusters (Figure 1B). Based on canonical marker genes (Supporting Information S1: Table S2), 11 cell types were annotated, including tumor cells, CD8^+^ T cells, CD4^+^ T cells, fibroblasts, macrophages, endothelial cells, epithelial cells, stromal cells, monocytes, vascular smooth muscle cells, and glial cells (Figure 1C).

**FIGURE 2:**
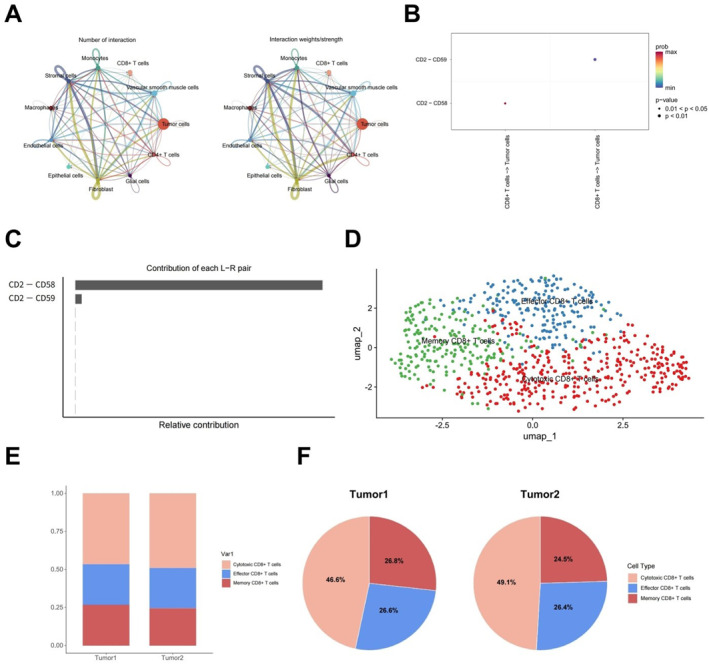
Cell–cell communication and T cell subtype analysis. (A) Cell–cell communication network in breast cancer brain metastasis; (B) heatmap of the CD2/CD58 axis showing intercellular communication intensity; (C) the relative contribution of L–R pairs to cellular interactions; higher values on the *x*‐axis indicate a greater role of the corresponding L–R pair in mediating intercellular signaling; (D) annotation of CD8^+^ T cell subtypes; (E) bar chart showing the proportions of CD8^+^ T cell subpopulations; and (F) pie charts representing the percentage composition of the three CD8^+^ T cell subsets.

Next, we analyzed the cell–cell communication network between CD8^+^ T cells and other cell types. CD8^+^ T cells exhibited strong interactions with tumor cells, fibroblasts, endothelial cells, and macrophages (Figure 2A). Notably, the CD2–CD58 interaction, where CD2 is expressed by T cells and CD58 by tumor cells, was significantly elevated, suggesting a key immunoregulatory function (Figure 2B). Contribution analysis of different cell types within the CD2–CD58 signaling pathway (Figure 2C) further confirmed the central role of CD8^+^ T cells and tumor cells in this signaling interaction.

CD8^+^ T cells were further classified into distinct subpopulations (Supporting Information S1: Table S3), including CTLs, CD8^+^ cytotoxic T cells, and memory‐like CD8^+^ T cells (Figure 2D). Subtype identity was confirmed based on the expression of representative marker genes such as *GNLY*, *NKG7*, *DUSP1,* *JUN, LTB, and COTL1* (Supporting Information S1: Figure S4). The results revealed a relatively low proportion of CTLs in tumor tissues, whereas CD8^+^ cytotoxic T cells were markedly enriched (Figure 2E). Specifically, CTLs accounted for approximately 26%, whereas CD8^+^ cytotoxic T cells comprised nearly 50% of both tumor samples (Figure 2F). The limited presence of CTLs may reflect their early engagement in antitumor responses, followed by depletion due to sustained antigen exposure and immunosuppressive signals within the TME. In contrast, chronic antigenic stimulation may favor the expansion of cytotoxic CD8^+^ T cells with enhanced tumor‐killing potential. These cells exhibit elevated expression of cytolytic effector molecules, such as PRF1 and GZMB, supporting their role in persistent tumor surveillance.

These findings highlight the functional heterogeneity of CD8^+^ T cell subsets in BCBM and underscore the importance of the CD2–CD58 axis in mediating T cell–tumor cell interactions. The enhanced signaling activity observed between effector T cells and tumor cells suggests that CD2–CD58 serves as a key regulatory pathway in sustaining T cell effector function and promoting antitumor immunity.

### 3.2 CD2 promotes CD8^+^ T cell proliferation and CTL activation

CD2 is a key regulator of CD8^+^ T cell proliferation and CTL activation, and its expression level critically influences CTL function.^40^ To elucidate the role of CD2, a targeted knockdown approach was employed for systematic functional assessment.

**FIGURE 3:**
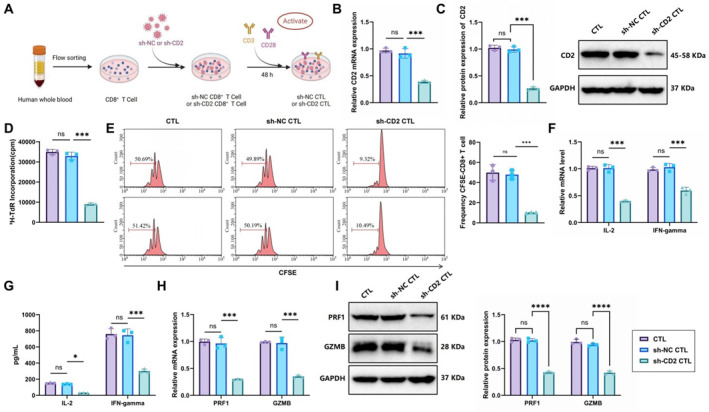
Effects of CD2 downregulation on CD8^+^ T cell proliferation and cytotoxic T lymphocyte function. (A) Workflow for CD8^+^ T cell isolation from peripheral blood of healthy donors and subsequent lentiviral transduction. Created in BioRender; (B, C) RT‐qPCR and western blot analysis of CD2 mRNA and protein expression in CD8^+^ T cells; (D) DNA synthesis was assessed using ^3^H‐TdR incorporation assay; (E) flow cytometry analysis of CFSE fluorescence intensity to evaluate cell cycle progression (two representative images per group shown); (F) RT‐qPCR analysis of IL‐2 and IFN‐γ mRNA levels; (G) ELISA quantification of IL‐2 and IFN‐γ in cell culture supernatants; and (H, I) RT‐qPCR and western blot analysis of PRF1 and GZMB expression in CD8^+^ T cells. All experiments were performed in triplicate. *p < 0.05, ***p < 0.001.

CD8^+^ T cells were isolated from the peripheral blood of healthy donors through flow cytometric sorting and subsequently transduced with either control (sh‐NC) or CD2‐targeting (sh‐CD2) lentivirus. After 48 h, cells were stimulated with anti‐CD3/CD28 antibodies to induce activation (Figure 3A). RT‐qPCR and western blot analysis confirmed stable CD2 expression in the sh‐NC CTL group, whereas a significant reduction in CD2 mRNA and protein levels was observed in the sh‐CD2 CTL group (Figure 3B,C).

In the CD8^+^ T cell proliferation assay, ^3^H‐TdR incorporation assay revealed that CD2 knockdown significantly suppressed DNA synthesis compared with sh‐NC controls (Figure 3D). Flow cytometry was used to evaluate CFSE fluorescence intensity as an indicator of cell cycle progression to further assess proliferative capacity changes. The results revealed a significant reduction in the proportion of actively dividing cells and a corresponding increase in cells remaining in the initial cycle phase in the sh‐CD2 CTL group, suggesting that CD2 downregulation impaired normal cell cycle progression, leading to limited proliferative ability (Figure 3E). To assess CTL activation status, we measured the expression of key cytokines IL‐2 and IFN‐γ. RT‐qPCR demonstrated a substantial reduction in IL‐2 and IFN‐γ transcripts following CD2 silencing (Figure 3F). In addition, ELISA analysis of cell culture supernatants showed significantly decreased protein secretion of both cytokines in the sh‐CD2 CTL group throughout the activation period compared to sh‐NC controls (Figure 3G). These findings indicate that CD2 downregulation severely impairs CTL activation and suppresses cytokine secretion, potentially compromising their effector function during immune responses. In addition, both RT‐qPCR and western blot analyses revealed significantly decreased mRNA and protein levels of PRF1 and GZMB in CD2‐deficient CTLs compared to control cells (Figure 3H,I), suggesting that CD2 knockdown not only weakens CTL activation but also significantly disrupts their cytotoxic function.

These findings highlight the critical role of CD2 in promoting CD8^+^ T cell proliferation and maintaining CTL activation and cytotoxic capacity.

### 3.3 CD2‐deficient CTLs suppress tumor cell apoptosis and promote proliferation, migration, and invasion

**FIGURE 4:**
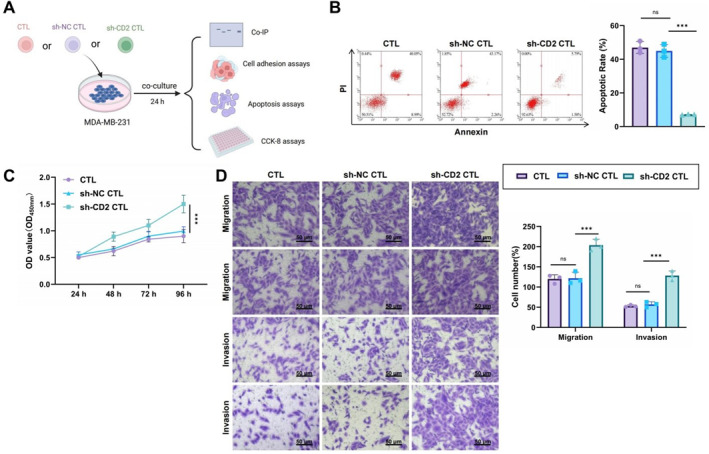
Effects of CD2 downregulation on CTL‐MDA‐MB‐231 interactions and tumor cell behavior. (A) Schematic of the coculture system involving CD8^+^ T cells and MDA‐MB‐231 cells. Created in BioRender; (B) apoptosis rate of MDA‐MB‐231 cells assessed using flow cytometry; (C) proliferation of MDA‐MB‐231 cells was evaluated using the CCK‐8 assay; (D) migration and invasion of MDA‐MB‐231 cells assessed using Transwell assays (two representative images per group shown). Scale bar: 50 μm. All cellular experiments were performed in triplicate. **p < 0.01, ***p < 0.001.

To simulate the metabolic interaction within the TME, CD8^+^ T cells from various experimental groups were cocultured with MDA‐MB‐231 breast cancer cells (Figure 4A). Western blot results confirmed that MDA‐MB‐231 cells exhibited low CD2 protein expression (Supporting Information S1: Figure S4C). Co‐IP analysis showed that the CD2–CD58 interaction between CTLs and MDA‐MB‐231 cells was significantly weaker in the sh‐CD2 CTL group compared to the CTL and sh‐NC CTL groups (Supporting Information S1: Figure S4D), suggesting that CD2 downregulation may impair the recognition of MDA‐MB‐231 cells using T cells. Further analysis using a CTL‐MDA‐MB‐231 adhesion assay revealed that the conjugation rate between T cells and MDA‐MB‐231 cells was reduced in the sh‐CD2 CTL group compared with the CTL and sh‐NC CTL groups (Table 1), indicating impaired CTL‐tumor cell binding due to CD2 silencing.

**TABLE 1:** Binding of CTL to MDA‐MB‐231 cells.

Group	% Conjugation (6 SD)	% Inhibition	Significance
CTL	34 ± 1		
sh‐NC CTL	36 ± 2	0	ns
sh‐CD2 CTL	12 ± 1	64	<0.05

We then evaluated the cytotoxic effects of CD2‐deficient CTLs on MDA‐MB‐231 cells. Flow cytometric analysis revealed a significantly lower apoptosis rate in MDA‐MB‐231 cells cocultured with sh‐CD2 CTLs compared to control groups (Figure 4B), suggesting that CD2 knockdown substantially impairs CTL‐mediated cytotoxicity. CCK‐8 assays further revealed a significant increase in MDA‐MB‐231 proliferation in the sh‐CD2 CTL group relative to the control groups (Figure 4C). Transwell migration and invasion assays confirmed that MDA‐MB‐231 cells cocultured with sh‐CD2 CTLs exhibited significantly higher migration and invasion abilities than those cocultured with CTL or sh‐NC CTL cells (Figure 4D), suggesting that CD2‐deficient CTLs may promote malignant tumor progression by reducing immune surveillance.

Together, these results demonstrate that CTLs mediate the recognition and adhesion to MDA‐MB‐231 cells through the CD2–CD58 axis, thereby influencing tumor cell apoptosis, proliferation, migration, and invasion.

### 3.4 CD2‐deficient CTLs promote brain metastasis in a breast cancer mouse model

**FIGURE 5:**
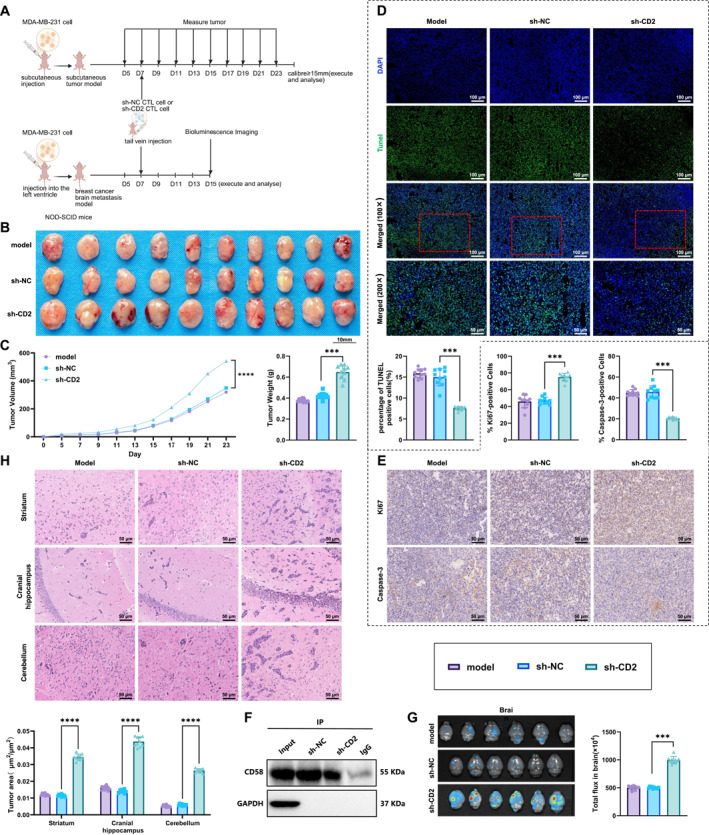
Impact of CD2‐deficient CTLs on breast cancer brain metastasis and tumor growth. (A) Experimental workflow of subcutaneous tumor and brain metastasis models. Created in BioRender; (B, C) tumor volume and weight measurements in the subcutaneous tumor model; (D) TUNEL assay for detection of apoptotic cells in tumor tissues. Scale bar: 100 μm; (E) IHC analysis of Ki67 and cleaved Caspase‐3 expression in tumor tissues. Scale bar: 50 μm; (F) Co‐IP analysis of CD2‐CD58 interactions in tumor tissues; (G) BLI and quantification of photon flux in the brain metastasis model; and (H) hematoxylin and eosin staining and quantification of metastatic tumor area in brain tissues. Scale bar: 50 μm. *N* = 10. ***p < 0.001.

To further investigate the effect of CD2‐deficient CTLs on BCBM, we established both subcutaneous and brain metastasis models in mice to evaluate their impact on primary tumor growth and metastatic progression (Figure 5A).

In the subcutaneous tumor model, mice in the sh‐CD2 CTL group exhibited significantly increased tumor volume and weight compared to the sh‐NC CTL group (Figure 5B,C). TUNEL staining revealed that the proportion of apoptotic cells in tumor tissues was significantly reduced in the sh‐CD2 CTL group relative to controls (Figure 5D, Supporting Information S1: Figure S5A). The IHC analysis further demonstrated that the percentage of Ki67‐positive cells (a marker of cell proliferation) was significantly higher in the sh‐CD2 CTL group, whereas the expression of Caspase‐3 (a proapoptotic marker) was markedly lower, indicating that CD2‐deficient CTLs promoted tumor cell proliferation and suppressed apoptosis (Figure 5E, Supporting Information S1: Figure S5B). Coimmunoprecipitation analysis of tumor tissues revealed weakened CD2–CD58 interactions in the sh‐CD2 CTL group, as indicated by reduced CD58 band intensity (Figure 5F).

In the BCBM model, BLI indicated that CTLs with low CD2 expression enhanced the brain metastatic spread of MDA‐MB‐231 cells following intracardiac injection (Figure 5G). H&E staining confirmed that the brain metastatic lesion area was substantially larger in the sh‐CD2 CTL group than in the sh‐NC CTL group (Figure 5H, Supporting Information S1: Figure S5C).

Collectively, these findings demonstrate that CD2‐deficient CTLs promote breast cancer progression and brain metastasis by disrupting CD2–CD58 interactions.

### 3.5 CD2‐deficient CTLs suppress the urea cycle and enhance pyrimidine biosynthesis in MDA‐MB‐231 cells and a BCBM mouse model

**FIGURE 6:**
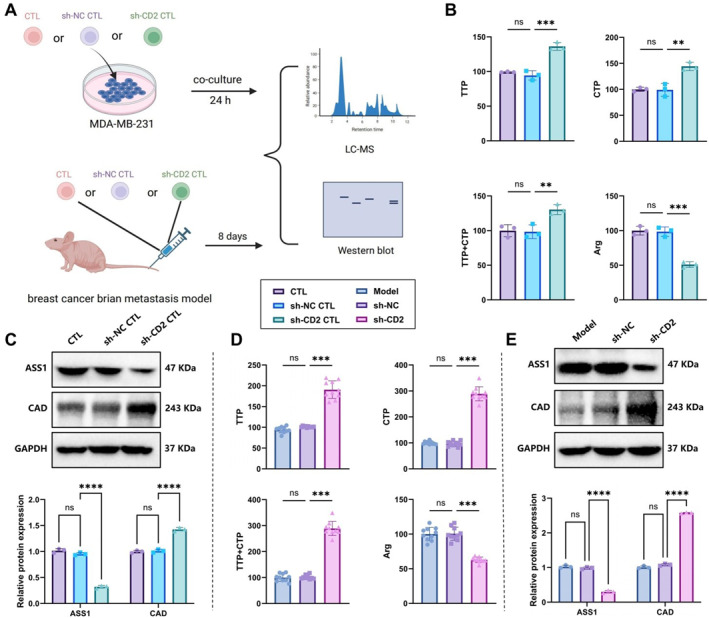
Effects of CD2‐deficient CD8^+^ effector T cell on urea cycle and pyrimidine metabolism in breast cancer cells and brain metastasis models. (A) Schematic illustration of metabolic markers associated with the urea cycle and pyrimidine biosynthesis. Created in BioRender; (B) LC–MS analysis of pyrimidine metabolites (TTP, CTP) and Arg levels in MDA‐MB‐231 cells from coculture experiments; (C) western blot analysis of ASS1 and CAD protein expression in MDA‐MB‐231 cells after coculture; (D) LC–MS detection of TTP, CTP and Arg levels in tumor tissues from the breast cancer brain metastasis mouse model; and (E) western blot analysis of ASS1 and CAD protein expression in tumor tissues. *p < 0.05, **p < 0.01, ***p < 0.001.

To investigate the impact of CD2 downregulation on tumor cell metabolism, we analyzed changes in pyrimidine metabolism and urea cycle‐related molecules in MDA‐MB‐231 cells and tumor tissues from BCBM mice using LC‐MS and western blot (Figure 6A).

In the in vitro coculture experiments, levels of TTP, CTP, and TTP + CTP in MDA‐MB‐231 cells were elevated in the sh‐CD2 CTL group compared with the CTL and sh‐NC CTL groups, whereas Arg levels were markedly reduced (Figure 6B). Western blot results showed that expression of ASS1 (a key urea cycle enzyme) was significantly decreased, whereas expression of CAD (a key enzyme complex in pyrimidine biosynthesis) was significantly increased in the sh‐CD2 CTL group (Figure 6C).

In the in vivo experiments, tumor tissues from sh‐CD2 group mice showed significantly higher TTP, CTP, and TTP + CTP levels, along with a pronounced reduction in Arg levels compared to the sh‐NC group (Figure 6D). Western blotting further validated a marked decrease in ASS1 protein levels and a concomitant upregulation of CAD expression in tumor tissues derived from the sh‐CD2 group (Figure 6E).

These findings indicate that CD2‐deficient CTLs suppress the urea cycle and enhance pyrimidine biosynthesis in both MDA‐MB‐231 cells and tumor tissues of BCBM mice. This suggests that the CD2–CD58 axis may influence the brain metastatic microenvironment of breast cancer by modulating tumor cell metabolic reprogramming.

## 4 DISCUSSION

The study provides a systematic characterization of the CD2–CD58 signaling axis as a key regulator in BCBM. The findings demonstrate that this axis facilitates both microenvironmental remodeling and metastatic progression by modulating CTL function and tumor metabolic reprogramming. Single‐cell transcriptomic analysis identified a high level of CD2–CD58 axis activity between CTLs and tumor cells. Functional experiments further confirmed that CD2 deficiency significantly impaired CTL proliferation, activation, and cytotoxic capacity while enhancing tumor cell proliferation, migration, and immune evasion. CD2 also influenced metabolic pathways, such as pyrimidine biosynthesis and the urea cycle via CTL‐tumor cell interactions, suggesting its involvement in tumor metabolic reprogramming. This study is the first to establish a mechanistic link between CD2, T cell function, and metabolic regulation, proposing CD2 as a critical node bridging immune responses and metabolism, with significant scientific and translational implications.

In the context of immune‐tumor interactions, the activation status and cytotoxicity of CTLs are central to antitumor immunity.^41^, ^42^ Previous studies have implicated CD2 as a costimulatory molecule that enhances early TCR signaling and promotes IL‐2 secretion, although most evidence originated from basic immunological systems without validation in solid tumor settings.^43^, ^44^ CD2 knockdown markedly reduced CTL proliferation, expression of activation markers (IL‐2 and IFN‐γ), and cytotoxic effectors (PRF1 and GZMB), indicating that CD2 plays a critical role not only in T cell adhesion but also in sustaining CTL functionality. Furthermore, reduced CD2 expression weakened CTL‐mediated recognition and induction of apoptosis in breast cancer cells while promoting tumor proliferation and migration. The findings suggest that CD2 plays a pivotal role in initiating T cell activation and maintaining effective antitumor responses, highlighting its potential as a therapeutic target for overcoming immune tolerance in BCBM and potentially other solid tumors.

Metabolic reprogramming has recently emerged as a key driver of tumor progression. The competition between tumor and immune cells for metabolic substrates has attracted increasing attention.^45^, ^46^, ^47^ Although previous studies have primarily focused on CTL exhaustion and its association with metabolic dysfunction, limited evidence addresses whether alterations in T cell functional states can, in turn, influence tumor cell metabolism.^48^, ^49^, ^50^ The present study provides the first evidence that CD2 downregulation impairs CTL function and actively reshapes tumor metabolism. In both coculture and in vivo models, CD2‐deficient CTLs led to downregulation of ASS1 and depletion of Arg, indicating suppression of the urea cycle. Concurrently, key enzymes involved in pyrimidine synthesis, including CAD and CTP, were upregulated, enhancing nucleotide production. This metabolic shift favors tumor cell proliferation and contributes to a more immunosuppressive microenvironment. Based on these findings, an “immune regulation–metabolic feedback” model is proposed to provide a new framework for understanding tumor–immune interactions within the TME.

By establishing a CD2‐knockdown CTL‐MDA‐MB‐231 coculture system and validating results in both subcutaneous and brain metastasis mouse models, we comprehensively evaluated the role of CD2 in tumor immune surveillance. Our in vivo results showed that CD2‐deficient CTLs significantly accelerated tumor growth and metastasis, as evidenced by increased subcutaneous tumor volume, enlarged brain metastatic lesions, elevated Ki67‐positive proliferating cells, and reduced Caspase‐3 expression, indicating enhanced tumor proliferation and reduced apoptosis. In contrast, CTLs with intact CD2 expression exhibited stronger antitumor activity, reinforcing the critical role of CD2 in maintaining CTL‐mediated tumor control. Consistency between in vitro and in vivo results strengthens the reliability and translational potential of the findings. Unlike previous studies limited to single cell lines or short‐term systems, the multidimensional design of the current study enhances clinical relevance.

Compared with conventional bulk RNA sequencing, scRNA‐seq offers distinct advantages in dissecting cell‐cell communication and revealing the dynamic changes in the immune microenvironment.^51^, ^52^, ^53^ In this study, we utilized the publicly available GSE186344 dataset from the GEO database and applied tools, such as CellChat, to analyze signaling interactions. High expression and interaction activity of CD2 and CD58 between CTLs and tumor cells in BCBM were identified, supporting downstream experimental validation and providing molecular evidence for the regulatory role of the CD2–CD58 axis in immune responses and tumor progression. Furthermore, subpopulation analysis at the single‐cell level demonstrated that CTL subsets with high CD2 expression exhibited enhanced metabolic activity and cytotoxic potential, underscoring the pivotal role of CD2 in functional heterogeneity among T cell subsets. Our study presents a comprehensive and credible mechanistic framework integrating bioinformatics and experimental validation.

Immunotherapy for breast cancer continues to face significant limitations, including low response rates and frequent resistance, particularly in patients with brain metastases where treatment efficacy remains limited.^54^, ^55^, ^56^ Existing immune‐based strategies primarily target immune checkpoints, such as PD‐1/PD‐L1 and CTLA‐4, whereas adhesion and costimulatory signaling pathways remain underexplored.^57^, ^58^, ^59^ The present study identifies CD2 as a modulator of T cell activation and a potential mediator of metabolic interaction within the TME, offering a novel dimension for immune intervention in BCBM. CD2 may serve as a biomarker for assessing immune competence or act synergistically with checkpoint inhibitors to enhance antitumor immunity. Restoration of CD2 function could offer a promising strategy to overcome immune tolerance in brain metastases and merits further clinical investigation. Moreover, this study reveals that the CD2–CD58 axis regulates the interplay between CTL function and tumor metabolism through multiple mechanisms. CD2 downregulation not only directly impairs CTL activation and cytotoxicity but may also indirectly relieve the suppression of CAD expression in tumor cells by reducing IFN‐γ secretion, thereby promoting pyrimidine synthesis. In addition, impaired CTL function leads to reduced competition for arginine metabolism, potentially allowing tumor cells greater access to arginine for polyamine synthesis, which may further drive malignant progression. These findings offer a novel perspective on the role of immune–metabolic crosstalk in BCBM, although the specific molecular mechanisms require further investigation.

In summary, this study systematically reveals the pivotal role of the CD2–CD58 signaling axis in BCBM, demonstrating that CD2 downregulation impairs CTL function, disrupts immune recognition, and promotes tumor metabolic reprogramming, ultimately facilitating tumor progression and microenvironmental remodeling. These findings offer novel mechanistic insight into BCBM by linking immune dysfunction with metabolic rewiring. From a scientific standpoint, the work addresses a critical gap in understanding the regulatory function of CD2 in CTL‐mediated immunity and immune–metabolic crosstalk in solid tumors, such as BCBM, expanding current immunological theory. Clinically, CD2 emerges as a potential candidate for risk stratification, immune phenotyping, and targeted intervention in BCBM, providing new strategies for precision oncology. Nonetheless, several limitations remain. Functional validation was primarily performed using murine models and in vitro systems, with no direct corroboration from clinical patient samples. Second, although the role of the CD2–CD58 axis has been identified, its universality across different tumor subtypes and immune microenvironments remains to be fully elucidated. Future research should incorporate clinical tissue specimens and explore the function of CD2 across diverse T cell subsets, thereby advancing the translational potential of CD2‐targeted strategies in metastatic breast cancer and paving the way for immune‐metabolic‐based interventions in clinical settings.

## 5 CONCLUSION

The study identifies the CD2–CD58 signaling axis as a critical immunoregulatory mechanism within the microenvironment of BCBM. High CD2 expression enhances CTL recognition and cytotoxicity toward tumor cells, whereas CD2 downregulation suppresses CTL proliferation and effector function, compromises immune surveillance, and promotes tumor cell proliferation, migration, and invasion. Moreover, CD2 deficiency disturbs the urea cycle and pyrimidine biosynthesis, contributing to metabolic reprogramming that accelerates tumor progression and brain metastasis.

This work carries both scientific and clinical implications. From a scientific perspective, the integration of single‐cell multiomics analysis provides new insight into the interplay between immune regulation and metabolic rewiring. From a clinical standpoint, CD2 represents a promising immunotherapeutic target, particularly in the development of precision strategies for managing BCBM. Several limitations should be noted. The experimental design predominantly relies on murine models and in vitro systems, lacking direct validation using patient‐derived samples. Moreover, the generalizability of CD2–CD58 signaling across distinct tumor subtypes and immune landscapes remains to be fully clarified. Future studies should incorporate clinical tissue specimens and evaluate CD2 function across diverse T cell subsets to support the translational relevance of CD2‐targeted interventions. In addition, systematic dissection of the upstream and downstream regulatory networks of the CD2–CD58 axis, as well as its potential synergy with current immune checkpoint inhibitors, may provide deeper mechanistic understanding and broaden the therapeutic scope of immune‐based strategies for BCBM.

## AUTHOR CONTRIBUTIONS

Guanyou Huang conceived and supervised the study. Yigong Wei, Xiaohong Hou, and Xin Jia performed experiments and data collection. Yong Yu and Xu Li conducted bioinformatics and statistical analyses. Shanshan Yu contributed to in vivo experiments and data interpretation. Guanyou Huang and Yigong Wei drafted the manuscript. All authors have reviewed and approved the final version of the manuscript.

## CONFLICT OF INTEREST STATEMENT

The authors declare no conflicts of interest.

## ETHICS STATEMENT

All animal experiments were conducted in accordance with protocols approved by the Animal Care and Use Committee of The Second People's Hospital of Guiyang (Jinyang Hospital) (Approval No. 2403172).

## CONSENT FOR PUBLICATION

Not applicable.

## Supporting information

Supporting Information S1

## Data Availability

All data generated or analyzed during this study are included in this article and/or its supplementary material files. Further inquiries can be directed to the corresponding author.
