# Supplementary material for: CD2–CD58 axis orchestrates cytotoxic T lymphocyte function and metabolic crosstalk in breast cancer brain metastasis
Source: J Cell Commun Signal. 2025 Aug 24;19(3):e70040. doi: 10.1002/ccs3.70040 (PMC12375503; doi:10.1002/ccs3.70040)
Supplement: Supplementary file 1 — Supporting Information S1 [file CCS3-19-e70040-s001.docx]

**Table S1. RT-qPCR primer sequence (human).**

| **Gene ID** | **Forward Primer (5′-3′)** |
| --- | --- |
| GAPDH-F | 5′-GAAGGTGAAGGTCGGAGTCA-3′ |
| GAPDH-R | 5′-CATGGGTGGAATCATATTGGAA-3′ |
| CD2-F | 5′-TCAAGAGAGGGTCTCAAAACCA-3′ |
| CD2-R | 5′-CCATTCATTACCTCACAGGTCAG-3′ |
| PRF1-F | 5′-GGCTGGACGTGACTCCTAAG-3′ |
| PRF1-R | 5′-CTGGGTGGAGGCGTTGAAG-3′ |
| GZMB-F | 5′-CCCTGGGAAAACACTCACACA-3′ |
| GZMB-R | 5′-GCACAACTCAATGGTACTGTCG-3′ |

Note: F: forward; R: reverse.

**Table S2. Cell types and marker genes.**

| **Cell Type** | **Marker gene** |
| --- | --- |
| Tumor cells | SLC39A6, AGR2 |
| Vascular smooth muscle cells | HIGD1B, RGS5 |
| CD8^+^ T cells | CCL5, NKG7 |
| Stromal cells | CCDC102B, GGT5 |
| Monocytes | S100A8, IL1B |
| Epithelial cells | CALML5, AARD |
| Endothelial cells | CLDN5, ADGRL4 |
| Macrophages | SPP1, TREM2 |
| Glial cells | PLP1, CTHRC1 |
| CD4^+^ T cells | LTB, TRAC |
| Fibroblast | CTHRC1, ISLR |

**Table S3. Annotation of T cell subtypes.**

| **Cell Type** | **Marker gene** |
| --- | --- |
| CD8^+^ Effector T | SLC39A6, AGR2 |
| CD8^+^ Cytotoxic T cells | DUSP1, JUN |
| CD8^+^ Memory T | LTB, COTL1 |


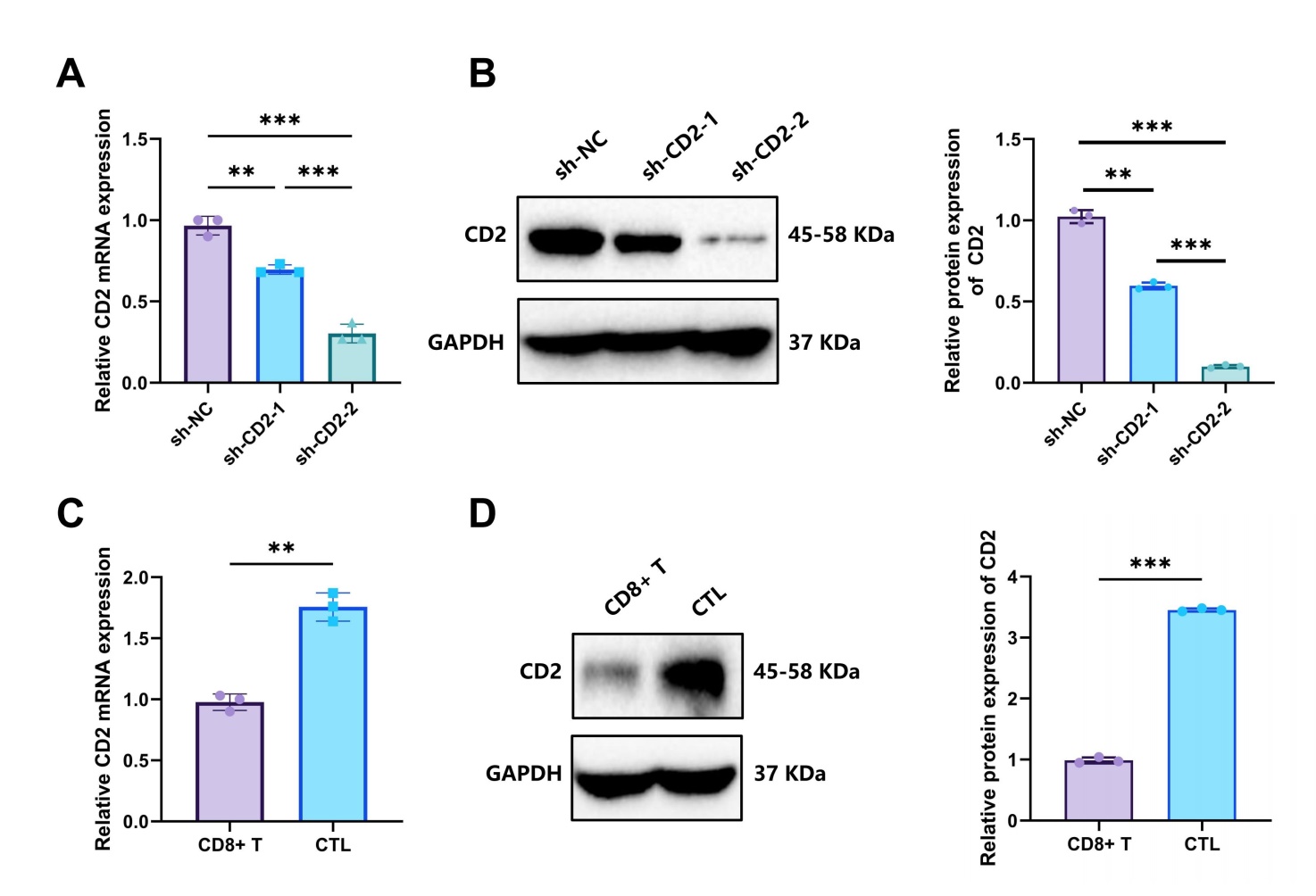


**Figure S1. Validation of CD2 knockdown efficiency and CD8⁺ T cell activation.**Note: (A) RT-qPCR analysis of CD2 mRNA expression in CD8⁺ T cells after shRNA lentiviral infection; (B) Western blot analysis of CD2 protein expression in CD8⁺ T cells after infection; (C) RT-qPCR analysis of CD2 mRNA expression in CD8⁺ T cells following anti-CD3/CD28 activation; (D) Western blot analysis of CD2 protein expression in CD8⁺ T cells following activation. All cellular experiments were performed in triplicate. **p* < 0.05, ***p* < 0.01, ****p* < 0.001.


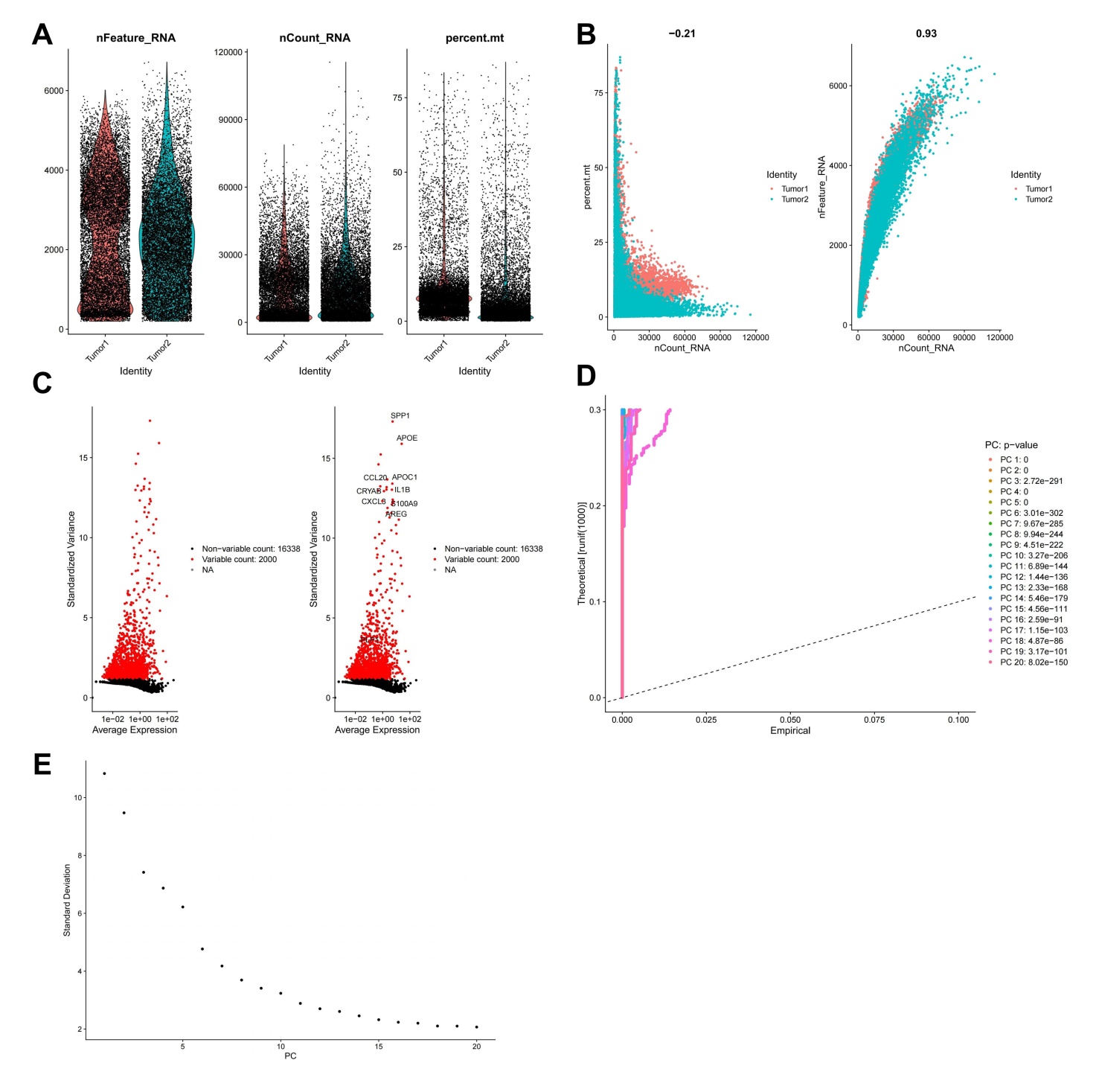


**Figure S2. Quality control and variance analysis of single-cell transcriptomic data.**Note: (A) Quality control metrics of scRNA-seq data from two tumor specimens, showing the distribution of nFeature_RNA, nCount_RNA, and percent.mt per cell; (B) Correlation analysis between nCount_RNA and percent.mt (left), and between nCount_RNA and nFeature_RNA (right); (C) Identification of HVGs: red dots indicate HVGs, while black dots represent non-variable genes; (D) JackStrawPlot illustrates the distribution of *p*-values for each PC; (E) ElbowPlot determines the optimal number of PCs for downstream analyses based on the inflection point of variance explained.


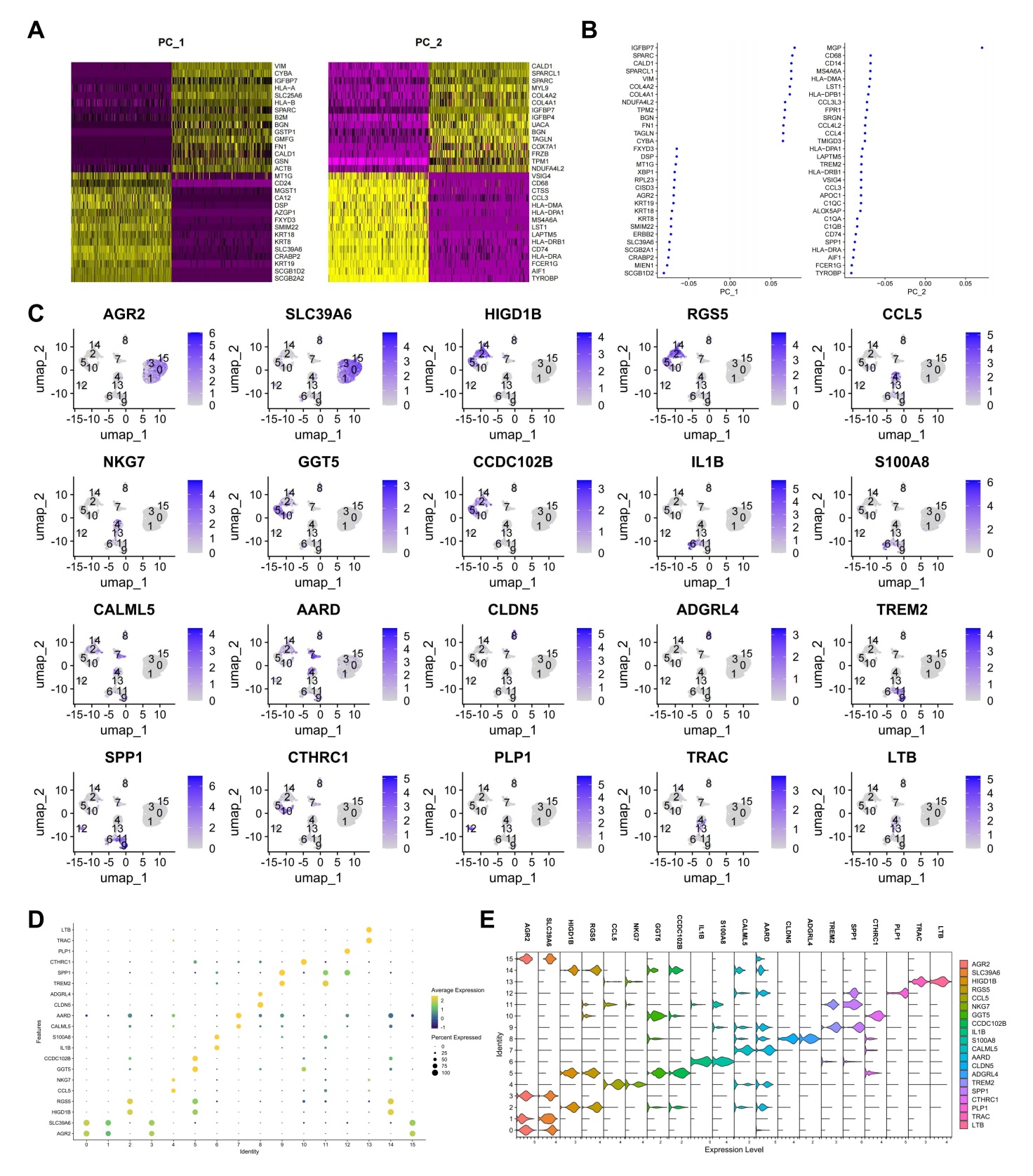


**Figure S3. Visualization of marker gene expression in BCBM.**Note: (A) Heatmap of gene expression in the top two PCs; (B) Dot plot of gene composition in the top two PCs; (C) Feature plot showing the expression levels of cell-type marker genes (darker color indicates higher expression); (D) Bubble plot of marker gene expression (larger circles represent higher expression percentages); (E) Violin plots of marker gene expression levels across different cell clusters.


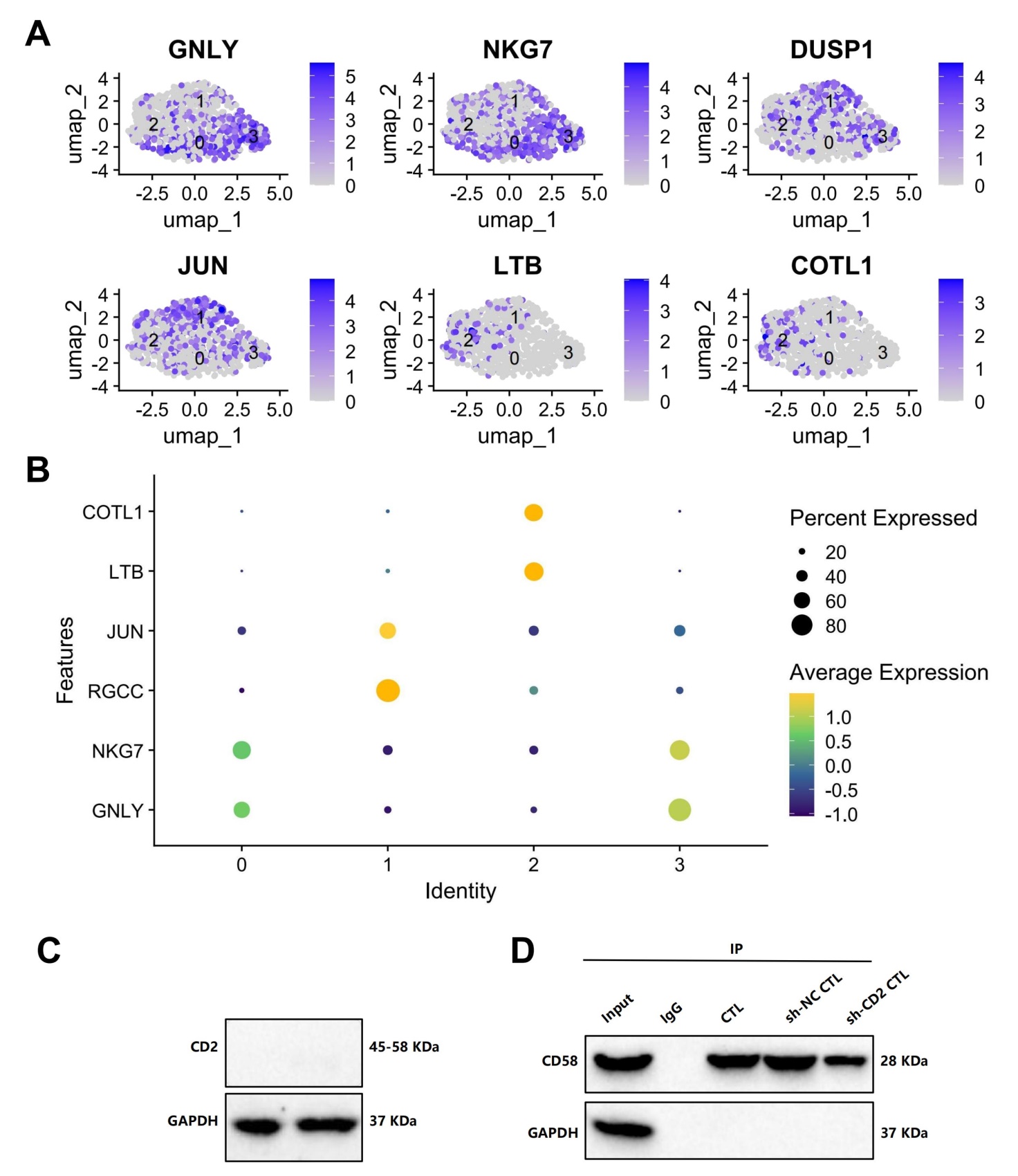


**Figure S4. Annotation of T cell subtypes.**Note: (A) Feature plot of T cell marker gene expression (darker color indicates higher expression levels); (B) Bubble plot showing the expression proportion of T cell marker genes (larger bubbles indicate higher proportions); (C) Western blot analysis of CD2 expression in MDA-MB-231 cells; (D) Co-IP assay assessing the interaction intensity of CD2-CD58 between CTLs and MDA-MB-231 cells.


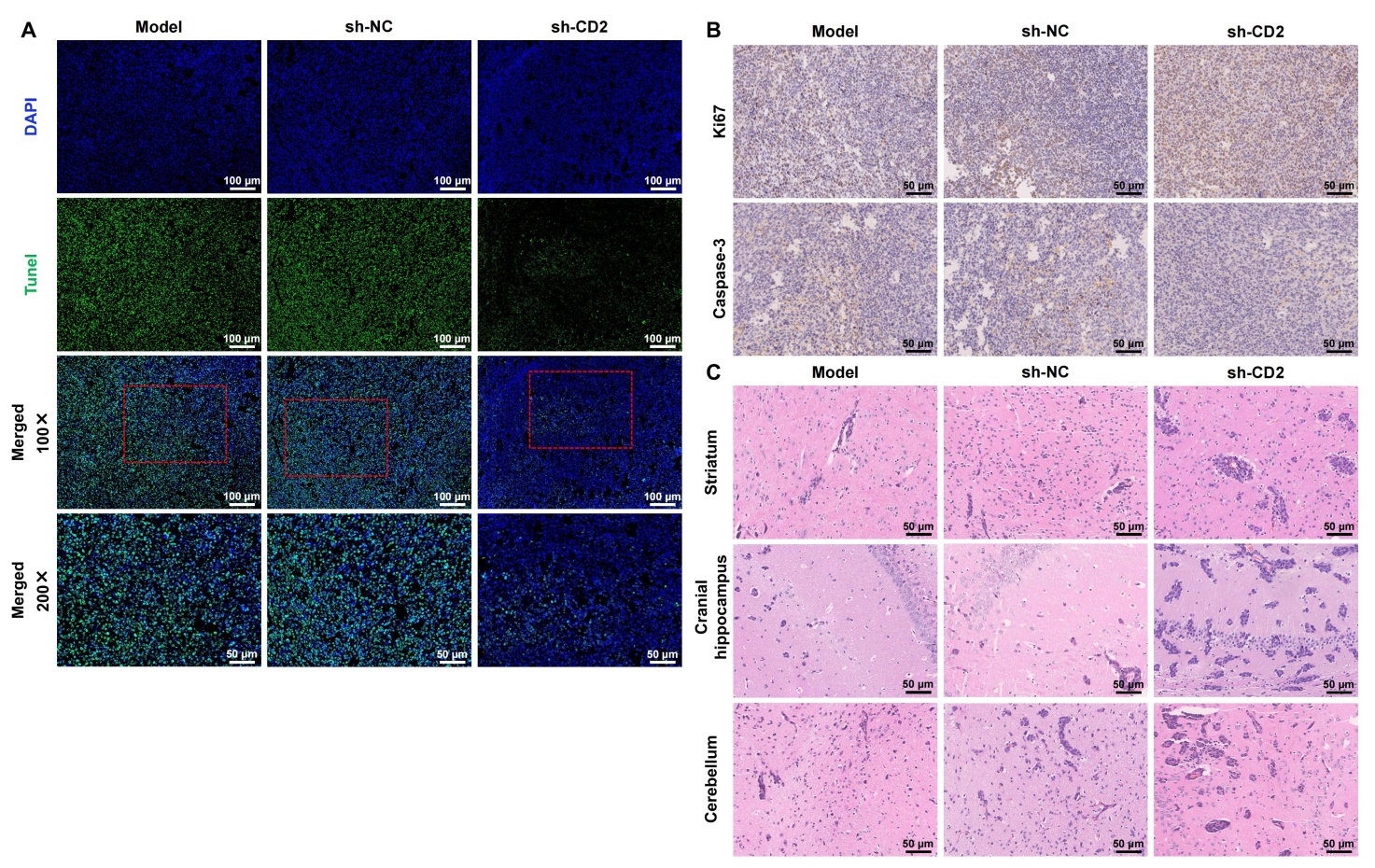


**Figure S5. Effects of CTLs with Low CD2 Expression on Breast Cancer Brain Metastasis and Tumor Growth.
(**A) TUNEL assay to detect the proportion of apoptotic cells in tumor tissues (second set of representative images shown); scale bar: 100 μm; (B) IHC to assess the expression and proportion of Ki67- and Caspase-3–positive cells in tumor tissues (second set of representative images shown); scale bar: 50 μm; (C) H&E staining of brain tissues from mice with breast cancer brain metastasis (two sets of representative images shown); scale bar: 50 μm.
